# From lunch breaks to late nights: a qualitative study of how workplaces can support healthy diet, physical activity and sleep behaviours in young adults

**DOI:** 10.1186/s12889-025-25595-8

**Published:** 2025-12-17

**Authors:** Cristiana Orlando, Zofia Bajorek, Alena Oxenham, Sally Wilson, Esther M. F. van Sluijs, Adam Martin, Hannah Fairbrother, Eleanor M. Winpenny

**Affiliations:** 1 Institute for Employment Studies, City Gate, 185 Dyke Rd, Brighton and Hove, BN3 1TL UK; 2 MRC Epidemiology Unit, Level 3 Institute of Metabolic Science, University of Cambridge School of Clinical Medicine, Cambridge, CB2 0SL UK; 3 Mohn Centre for Children’s Health and Wellbeing, School of Public Health, Imperial College London, London, UK; 4 Academic Unit of Health Economics, Leeds Institute of Health Sciences, University of Leeds, Leeds, LS2 9TJ UK; 5 School of Nursing and Midwifery, University of Sheffield, Barber House Annexe, 3a Clarkehouse Road, Sheffield, S10 2LA UK

**Keywords:** Employment, Diet, Physical activity, Sleep, Young adults

## Abstract

**Background:**

Early adulthood (age 16–24) is an important time for development of healthy behaviours such as diet, physical activity and sleep, which promote wellbeing and maintenance of a healthy weight. This study explored the perspective of young adult employees and employers on the influence of work and the workplace on these behaviours, and what employers and policy makers could do to support healthy behaviours among young adults in the workplace.

**Methods:**

Our research focused on four industries (food services, construction, early years education, and social care), comprising focus groups with young adult employees (aged 16–24) and with employers. Framework analysis of focus group transcripts compared findings and identified common themes across the different industries represented, with a focus on perceived challenges, solutions, and pathways to supporting healthy behaviours.

**Results:**

23 young adults and 28 employers took part in the research. Employers and young adults agreed that work and the workplace had a strong influence on health behaviours and health. Participants discussed both the negative influence of work, for example long working hours, poor working environments, but also the positive role of employers’ support for health, incentives for healthy behaviours and good relationships with managers. While young people recognised the role of employers in supporting their health, they deem that individuals are ultimately responsible. Employers primarily emphasise their role in providing education and raising awareness about healthy lifestyles.

**Conclusions:**

Structural, environmental and relational factors are all important in ensuring that workplaces support young people’s health.

**Supplementary Information:**

The online version contains supplementary material available at 10.1186/s12889-025-25595-8.

## Introduction

For many people, early adulthood (defined here as age 16-24y) is a time of life when they are at their most healthy. However, obesity prevalence increases faster during this period than at any other time during adulthood, with more than half the UK population living with overweight or obesity by their early thirties [1].Obesity is socially patterned, with higher BMI consistently seen among groups of lower socioeconomic position [2] and these inequalities in BMI increase through adolescence and adulthood [3]. Diet, physical activity and sleep are important risk factors for development of overweight and obesity [4], which also show inequalities by socioeconomic position [5]. These behaviours are often less healthy during adolescence and early adulthood [6–8] than in later life. The life transitions which take place during early adulthood, such as leaving the family home and starting employment contribute to changes in health behaviours [9], and may contribute to the development of social inequalities in behaviour which then persist throughout adult life [10]. Given this period of rapid change before behaviours become more stable, many have suggested that early adulthood should be considered a sensitive period for development of adult health behaviours [4, 11]. Interventions to support obesity-related health behaviours in early adulthood are considered to yield a “triple health benefit”: preventing immediate weight gain, enabling the development of habits that improve healthy behaviours across people’s whole adult life, and influencing the next generation [12].

Our health is strongly influenced by the people and environments around us [13], including our work environment. These environments may make both positive and negative contributions to health behaviours and health. Features of the workplace environment which may influence health behaviours include structural aspects such as working hours, distance to work, safety requirements, aspects of the physical environment like food availability, and the social environment, for example, the influence of colleagues [14, 15]. There is considerable literature across adult ages around workplace interventions aiming to improve health behaviours and prevent weight gain, including both individual-level [16] and workplace environment interventions [17], but to date there has been little focus on young adult employees, a group of high policy concern. Many studies focus on cognitive interventions including education or counselling [16] rather than low agency interventions like provision of healthy food [17] despite previous research suggesting that interventions requiring lower individual agency may be most effective in low socioeconomic [18] and younger age groups [19]. Although 63% of young adults are in full-time or part-time employment [17], little is known about the influence of the work environment on health behaviours and health in young adults.

Our study aimed to understand, from the perspective of young adult employees and employers in sectors with a high proportion of young adult employees, the role of the workplace in supporting healthy behaviours. We sought to answer the following research questions:

1. What is the influence of work and the working environment on health and health behaviours among young adults?
2. What could employers and policy makers do to support better diet, physical activity and sleep behaviours among young adults in the workplace?

## Methods

### Study design

A generic qualitative research design was taken in line with our flexible, pragmatic approach to answering our research questions [20]. Reporting follows the Consolidated Criteria for Reporting Qualitative Research (COREQ) to ensure a thorough explanation of our study design and therefore promote the transferability of our study [21, 22]. We focussed our research on four industries which have a high proportion of young adult employees, high levels of social deprivation in their workforce (including high levels of semi-routine/routine occupations in the social care sector, and low levels of higher education in the construction sector) and represent a diverse range of workplace experiences: food services, construction, early years education, and social care (the provision of personal, practical and emotional support to vulnerable individuals) (Supplementary Table 1). Two different sets of focus groups were conducted, with young adults and with employers. ZB and CO facilitated the focus groups, both female researchers with experience in workforce health and wellbeing and obesity (ZB) and young people, health and work (CO). Each young adult focus group lasted 1.5 h, and each employer focus group 1 h. All focus groups were conducted online using Microsoft Teams, and were facilitated through the use of semi-structured discussion guides (Supplementary Material). Each focus group was aided by the use of Google Jamboard, an interactive whiteboard, where participants were able to record their thoughts on the questions discussed in the focus group anonymously. The study received ethical approval from the University of Cambridge Humanities and Social Sciences Ethics Committee [reference 23.348, 22Nov2023].

### Sample recruitment

Young people were eligible to take part in the research if they were aged 16-24y, not in full-time education^1^, and working in one of the four industries (social care, food services, early years education and care, and construction) in the UK. Employers eligible for the research were those in managerial or HR positions, who had both an understanding of organisational policies and practices, as well as good oversight of what is happening in practice on the ‘employee front-line’.

In this study we conducted four focus groups for young adults, and four for employers, one across each industry sector. Each focus group included 5–8 participants [23]. This number afforded the opportunity for in-depth and lively discussion, exchange of ideas, and social support among participants, without feeling too overwhelming for participants or difficult to manage for facilitators, particularly in an online context [24]. The focus group facilitators (ZB and CO) had no prior relationship with the research participants.

Young people were sampled to represent a diverse group of participants across ethnic groups, the 16–24 age range, and full/part-time employees, drawing on analysis of the Labour Force Survey (LFS) for each of the selected industries, to ensure a representative sample. Employers were sampled based on the sectors that young people were drawn from, with additional criteria including geography and other characteristics such as company size and employer role. In this way, we purposively sampled to ensure a relevant range of participants.

Research participants were recruited through Roots, a market research agency, following a screening questionnaire to ensure they met the eligibility criteria for the research. Given the strict sampling and eligibility criteria, research participants were targeted and invited to take part in the research. Young people were offered a shopping voucher as an incentive and as a recognition of the time they gave for the research. Informed consent was obtained from all participants, prior to research participation. Participants were given an information sheet about the research and signed a consent form. The research was undertaken in accordance with the World Medical Association Declaration of Helsinki.

### Data analysis

The focus groups were audio recorded and transcribed. The research team used Google Jamboard to mobilise discussion and engagement, but only the focus group transcripts were used for data analysis. Framework Analysis [25, 26] was adopted to compare findings and identify common themes across the different industries represented. While researchers mobilised a coding framework based on the topic guide (a deductive approach), we also sought to identify emerging themes through close reading of the transcripts (an inductive approach). Our findings, however, mapped well onto our initial scaffolding framework with a focus on perceived challenges, solutions, and pathways to better health-relevant behaviours. Designed specifically for analysing qualitative data in applied policy research, framework analysis afforded a structured, organized method of identifying, describing and interpreting key patterns within and across cases [25]. We followed the five step approach of: (i) data familiarization (developing an initial understanding of the data) (CO, ZB, EW, HF), (ii) identification of an analytic thematic framework (moving from broad descriptions of the data to developing detailed understandings) (HF), (iii) indexing (initially testing and then applying the framework to the study data) (CO, HF), (iv) charting (developing matrices summarizing the study data (CO, HF) and (v) mapping and interpretation (revisiting and reviewing earlier steps and formulating a coherent ‘story’ about the structure and patterns within the data) (CO) [26]. The researcher team met to revisit, review and discuss at each stage of the five-step approach. These meetings enabled the team to: develop a shared understanding; reflect on the influence of our different professional backgrounds – workforce health and wellbeing and obesity (ZB), young people, health and work (CO) and young people and health inequalities (HF) and to share and check a small sample of each other’s work to ensure inter-rater reliability and consensus throughout the analysis process.

## Results

In total, 23 young adult employees and 28 employers took part in the research (an overall sample of 51 participants). The sociodemographic breakdown for the sample of employers and young adult employees is shown in Table 1. It should be noted that more females than males took part in the research - this is reflective of the wider make-up of several of the industries selected for the research. The four industries also differed in the sociodemographic characteristics of their workforce, as shown in Supplementary Table 1.

**Table 1:** Sociodemographic characteristics of employer and young adult focus group participants

		Employer focus group participants (*n* = 28)	Young adult focus group participants (*n* = 23)
Gender	Female	15	16
	Male	13	7
Age	19–21	Not reported	5
	22–25		18
Ethnicity	Black, Black British, Caribbean or African	4	3
	Asian or Asian British	3	4
	Mixed or multiple ethnic groups	1	3
	Prefer not to say	0	1
	White British	20	12
Industry	Construction	8	5
	Early Years	8	6
	Food Services	6	5
	Social Care	6	7
Business size	Less than 10	3	Not reported
	10–49	11	
	50–250	7	
	250–999	2	
	1000+	5	
Job title	Director	1	Not reported
	Manager	16	
	Owner	1	
	Senior Manager/Director	9	
	Supervisor	1	
Employment status	Full-time	Not reported	16
	Part-time		7

Our findings focus on five themes: the influence of work and the workplace on health behaviours; perceptions of the role of the employer; support for positive health behaviours already offered; suggestions for additional support; and discussion of wider aspects of health and wellbeing that influence health behaviours. These themes followed directly from the headings of the discussion guides that were used with young people and with employers, which were in turn developed from the research questions.

### The influence of work and the workplace on health behaviours

Across the four industries that were represented (construction, food services, early years, social care), young people agreed that their work strongly influenced their health behaviours. Participants reported experiencing stress, anxiety, and burnout related to their job responsibilities and work environments, often tied to long shifts, with impacts felt especially on dietary and sleep habits. For example, this included poor diet quality due to lack of time and poor quality of sleep due to stress. Young people highlighted that achieving a work-life balance was essential for wellbeing, but was often hindered by unsociable hours, particularly in the early years, food services and social care industries. In some industries, like construction and early years, the physically strenuous nature of the job and exposure to hazards were also raised as concerns in terms of the impact of work on health behaviours.

*“Good sleep*,* good water and nutritious food (…) at work we have one meal a day (…) but you can’t take a 5 minute break again.”* (young person, Food services).

*“I have worked in places where I was knocking down walls*,* it impacts me. You breathe in so much dust and rubbish*,* then don’t want to go to the gym”* (young person, Construction).

Employers shared the concerns expressed by young people, and emphasised the need for proper rest and downtime to maintain health and well-being. However, long hours and inadequate rest periods were also felt to be prevalent, often driven by financial necessity or job demands, leading to physical and mental strain. In particular, employers in the early years and social care sectors shared that given the physically and emotionally intensive nature of the job, employees are often too exhausted to look after their diet and physical health outside of work.

*“Work-life balance*,* you know not sticking to your hours but being balanced*,* starting work at a sensible time…we need to be conscious that you can’t expect people to consistently work overtime.”* (employer, Social care).

*“It’s not ideal to work two jobs because it is a lot and we don’t let any of our girls work over 48 hours with us (…) some work two jobs because that’s the way to make ends meet.”* (employer, Early years).

### Perceptions of the role of the employer in supporting positive health behaviours

Across all sectors, young people saw employers as playing an important role in supporting positive health behaviours. They mentioned the importance of good facilities and initiatives, such as providing healthy food options, encouraging exercise, and maintaining clean facilities, as well as having appropriate areas to take breaks. For example, in the early years sector, the provision of facilities like quiet, soundproofed break rooms and healthy meal options was deemed to significantly improve wellbeing. Additionally, young people felt that employers could play a role in supporting them to be active through incentives such as gym memberships and team bonding activities.

*“My organisation does really well (…) there are things like some discounts on healthy eating and vegan food brands (…) and they also have these blogs that encourage us to eat healthy*,* stay healthy*,* exercise*,* things like that.”* (young person, Social care).

*“You sometimes don’t have the right cleaning facilities [to wash up]*,* you don’t have enough breaks*,* and nowhere to heat up food.”* (young person, Construction).

There was a shared sentiment across young people that while employers have a role in supporting positive health behaviours, individuals are ultimately responsible. However, lack of sufficient breaks and poorly managed shifts were common challenges across many sectors, which hindered young people’s ability to manage their health. In the food services sector in particular, young people highlighted that employers could improve their practices in adhering to recommended rest periods and ensure adequate staffing to prevent overworking employees. They also suggested that providing healthier meal options and employers leading by example and fostering a culture of positive health habits could play an important role.

*“We’re all adults*,* we have to take our healthy habits and lifestyles as initiatives that come from ourselves. However*,* I do think that employers and companies should put like maybe incentives in place (…) it’s about making things available.”* (young person, Early years).

*“Yes but often the employers I worked for*,* they are not the healthiest people*,* they are also very stressed. There’s a weird contrast between being told “you should be healthy for your shift” and then being given a 10 hour shift with a short break in between.”* (young person, Food services).

On the other hand, employers primarily emphasised the importance of education and raising awareness. Employers suggested that young workers often enter the workforce without a clear understanding of what is reasonable and fair in terms of work expectations, and practices which can sustain wellbeing at work. In the food sector, for example, employers stressed the importance of educating staff on managing night shifts and having nutritious meals during shifts. Similarly to young people, employers generally agreed that modelling positive health behaviours is critical, as young workers are influenced by the actions and attitudes of their senior colleagues and managers.

*“We did a session on night working*,* ‘Night Club’ – spoke of loads of things people could do to be healthy*,* how to support sleep*,* brought in samples of food they could try.”* (employer, Food services).

*“Just have the opportunity to give them information and advice and suggestions.”* (employer, Social care).

However, when comparing findings from young people to those of employers, some tensions emerge around differing perceptions of these challenges. In terms of education, while welcomed, young people emphasized that improved working conditions need to be the foundation and precede any additional education. Further, while both young people and employers highlighted the importance of role models, young people reported they seldom saw positive role models in the workplace.

### Experiences of support for positive health behaviours in the workplace

Young people emphasised the quality of relationships with supervisors, mentors, and managers as a key aspect of the support they accessed. Across sectors, young people who reported positive interactions and open lines of communication felt that they were well supported at work and that this in turn helped them develop more positive health behaviours.

*“I have worked somewhere where I was working such long hours and it was taking a toll on me*,* my immune system was way down and I was stressed*,* I wasn’t sleeping and wasn’t eating right (…) but I had a good relationship with my boss and I said here are ways I am struggling*,* so they put a plan in for me.”* (young person, Early years).

However, in some sectors, young people perceived that there was little regard for their health and wellbeing, let alone interest in supporting positive health behaviours. In the food services sector, some young people reported a culture of normalising physical pain and injury. This was felt to discourage younger staff from seeking help or voicing their concerns, and was exacerbated by the widespread lack of sick pay, often forcing employees to work through illness. In the construction sector, young people reported that more senior colleagues disregarded PPE and safety guidelines, and that younger workers sometimes felt pressured to model these behaviours, with concerns raised around the long-term impact on physical health.

*“I got a burn yesterday*,* and one manager said ‘well I have this massive burn on my arm’ – like you’re only complaining because you’re younger. (…) I have a friend who broke his foot*,* was off for two days then the employer phoned in and asked him to come back and stay behind the bar.”* (young person, Food services).

Young people felt that in workplaces where these types of culture existed, little to no attention was paid to wider health behaviours, such as good nutrition, sleep, and exercise.

Across all sectors, employers tended to provide, to greater and lesser extents, access to some form of health support, with a greater focus on mental than physical health. These ranged from Employee Assistance Programmes (EAPs), Mental Health First Aid training for managers, resources from charities like Mind (a national mental health charity), as well as access to helplines and support from specific industry bodies (e.g. British Institute of Innkeeping). Some employers provided information on healthy eating; a small number provided healthy meal options. Overall, employers felt that the most effective type of support they could provide was creating a supportive workplace culture through good management, peer support, and friendly and open company cultures.

*“We try to make sure we’ve got material available for staff around stopping smoking (…) things like dry January*,* it’s about keeping people engaged*,* even if it is just a conversation.”* (employer, Early years).

*“We keep bakers for a very long time – we structure the rota so it’s long shifts*,* but very few during the week. We make sure people have a lot of space between shifts. It’s about not getting into people’s business*,* but taking responsibility to give them the opportunity to be rested.”* (employer, Food services).

### Suggestions for additional employment policy or workplace interventions to support positive health behaviours among young adults

Young people discussed what additional support they would like to see implemented in their workplace, and ideas for initiatives. The importance of encouraging a healthy diet was highlighted. Providing access to healthy food options, whether through free or subsidized meals, and ensuring the workplace had the right facilities (e.g. microwaves to heat up food) were key aspects. Some young people suggested offering cooking classes and education and information specifically around healthy eating.

*“They are dealing with healthy food all day for the children*,* so it would be nice if a little extra was made so we can have some as well. It’s also about feeling recognised by the managers.”* (young person, Early years).

Young people also called for better physical environments, such as adequate break areas, including spaces to sit and eat, and clean and functional facilities. Ensuring access to basic amenities like running water, hand sanitisers, clean toilets, and changing facilities was a common request, especially in more physically demanding sectors. It was felt that these facilities could encourage young people to bring in their own, healthier, meals and to exercise before and after work. Further suggestions included offering incentives for healthy behaviours, and formally recognising employees’ efforts and achievements, for example through reward and perk schemes, to both help boost morale and encourage embedded positive health habits.

*“Everything is individual*,* it depends on the site*,* but if there’s no basics you’ll want them. Running water on site*,* microwaves*,* fridge to keep food*,* so you can bring your own stuff.”* (young person, Construction).

Young people in each sector also provided suggestions specific to their sector. Among workers in the early years sector, young people called for protected break times, including more frequent and flexible breaks, and quiet spaces to take breaks, to ensure staff had enough time to eat their meals and to help manage fatigue. Young people in the food services sector highlighted the need to ensure at least one nutritious meal per shift. Finally, young people in social care raised the need for more flexible working arrangements to help balance heavy workloads and personal life, and reduce the impact of stress on sleep and energy levels.

*“Have a free meal provided*,* it should be a basic right. It’s nice to have at least one good proper meal. And having sick pay. [name of business] partnered up with PureGym so they offer us a discount on memberships*,* 20% off.”* (young person, Food services).

There was also a feeling that more could be done around health and wellbeing initiatives, from education (e.g. managing stress, nutrition, preventing burnout), to more practical measures such as hands-on cooking classes, and workplace initiatives to promote exercise and active living. Aside from these elements, there was also a shared sense that building peer networks and role modelling were effective and quick practices which could encourage better health habits among young workers.

*“We spoke a lot about food*,* but a lot of the time we need something physical. Like as a team*,* like a sport. We do lots of movement at work but it just strains your body rather than being movement for fun or mental health (…) find ways to bring team close without focusing on drinking*,* doing very physical things.”* (young person, Food services).

Among employers as well there were suggestions specific to each sector. In the early years sector, employers raised the challenge that small businesses often struggle to provide extensive healthcare and wellbeing packages due to financial constraints, and some employers highlighted an interest in seeking funding or grants to subsidise healthcare packages and gym memberships.

*“It is an undervalued industry (…) this would never happen but I would like some sort of funding or a grant to be put towards our staff and we want to show them that they are valued (…) it might be a gym membership or money towards lunches every day that are healthy.”* (employer, Early years).

Among employers in the food services sector, there was a shared view that young employees could benefit from nutrition education and cooking lessons, leveraging the expertise of in-house chefs. Implementing greater flexibility in work schedules was also seen as beneficial, as well as creating partnerships with other establishments to provide benefits and discounts supporting improved health behaviours around nutrition and exercise.

### Discussion of wider aspects of health and wellbeing that may influence health behaviours

Throughout the discussions, both young people and employers conceived of health and wellbeing broadly, and did not confine their discussions to health behaviours around diet, sleep, and physical activity alone, but rather addressed the breadth of factors which can indirectly impact these behaviours. In particular, mental health emerged as a major concern among young people across all sectors, with many describing experiencing stress, anxiety, and burnout from job responsibilities and work environments. They explained how the quality of relationships with bosses and colleagues and the overall work atmosphere significantly influenced both mental and physical health when negative. These issues had wider repercussions on young people’s behaviours around diet and exercise, as they described how they often lacked the energy or motivation to engage in healthier behaviours. Employers were also aware of widespread mental health issues, but often linked them to a perceived lower resilience to work stress among young people, potentially exacerbated by the Covid-19 pandemic’s impact on mental health and social skills.

*“If you have a boss stressing you out it impacts you*,* your mental health and sleep. If the boss gives me a hard time*,* it affects what I do outside of work.”* (young person, Construction).

*“I would say if I’ve had maybe like a challenging day where I’ve dealt with a lot of heavy topics*,* maybe someone in crisis and I don’t have an adequate debrief or a come down from that. That comes home with me and I want to order a takeaway and it’s that knock-on effect.”* (young person, Social care).

Employers, on the other hand, generally felt that financial concerns, tied to the low wages across the four sectors, significantly impact health behaviours among young employees. The high cost of healthy food, gym memberships, and wellness activities was felt to be a recurring issue amid a cost of living crisis. Employers also recognised a gap in financial knowledge among young people and agreed they could play a more significant role in financial education, as financial wellbeing was viewed as key for positive health behaviours. This included addressing concerns about taking sick days, understanding payslips, managing money, and signposting to financial advice.

*“I’ve had to talk through a pay slip because the girls have never had it before. They don’t understand NI (National Insurance)*,* pensions*,* and I have had to help them budget for the first time when they have moved out (…) I’ve helped them shop for cheaper things*,* and those little things that look after their wellbeing (…) wages are a big thing*,* and I always try and push the directors when they are down for everyone to get a pay rise because that makes a huge difference.”* (employer, Early years).

## Discussion

The findings outlined in the previous section report what employers and young people shared with the research team. The discussion aims to contextualise these findings and ground them in empirical evidence, providing a conceptual framework for interpretation. This study shows that both young people and employers in the industries of focus for this research (early years, construction, food services, social care) recognise that work and the work environment play a key role in supporting or impeding healthy behaviours among young adults. Across the discussions there was a recognition that structural factors such as pay and working hours had a strong impact on health behaviours. The findings also highlight some differences in emphasis between the two groups. Young employees emphasised the importance of environmental factors, such as the physical work environment and proactive initiatives from the employer to encourage positive health behaviours, and reported being receptive to employer initiatives. On the other hand, employers emphasised cognitive aspects, around improving education and supporting mental health, to support improved health behaviours around diet, sleep and exercise.

While our findings highlight that employers are actively trying to promote a healthy workplace, they also indicate concerns around resources and capacity to promote improved health behaviours, alongside uncertainty around what further steps to take. The current research points in the direction of a number of measures employers can consider to support health behaviours among employees, not all of which require large resource investments.

### Structural enablers of healthy behaviour

Both employers and employees in the research recognised that there are a number of structural factors which need to be in place first and as a basis for health behaviours. These factors include sufficient pay, manageable working hours and appropriate job design. Young adults and employers both highlighted the impact of low pay and long working hours on the ability to maintain health behaviours, with stress and tiredness from overworking contributing to poor diet and poor sleep outside of work. Employers also recognised the challenges for young people in relation to the cost of living and the need for some young people to work multiple jobs, which made it difficult for them to maintain healthy working hours. We know from extensive evidence that aspects of job quality, particularly pay, work intensity, and shift patterns, correlate strongly with health behaviours, particularly those tied to sleep [27–29] and nutrition [30–32]. This is true among all working populations working in low-paid sectors, and those with long or irregular shift patterns [33]. In particular, for young adults evidence has focused on the impact of working hours on healthy eating habits and on sleep quality, highlighting that young people working long hours experience more significant time-related barriers to healthy dietary intake [33, 34], and those working in service industries, where long shifts and irregular hours are common, have poorer sleep quality [35, 36].

### Environmental and cognitive interventions

When considering the role of the employer in supporting health behaviours, young people emphasised the use of environmental interventions such as provision of healthy food options and spaces to take breaks away from work. Environmental interventions can be complemented by cognitive interventions. These include some measures mentioned by employers in our research, such as education and information provision, for example through employers offering financial education, nutrition education, education around managing stress and sleep, as well as counselling, and positive role modelling. However, employers in the research tended to resort to cognitive measures as the first solution, but wider findings from young people and across the themes covered in the research, indicate that these measures are most effective when structural and environmental factors are addressed first. Our focus on sectors with high proportions of young people of low SEP is also relevant here as they are the most likely to be experiencing social deprivation and food insecurity, thus strengthening the need to address structural and environmental factors through, for example, access to nutritious meals [37].

These findings are in line with previous evidence, that to engage with many cognitive interventions requires a high level of individual agency [38], which may be limited in those working in jobs with long hours and poor pay [39, 40]. Existing evidence suggests that interventions which prioritise cognitive factors alone, i.e. aim to change knowledge or skills, tend to yield inconclusive results, at best [41], and widen inequalities, at worst [38]. More effective interventions are those with multiple components, which prioritise environmental changes in the workplace (e.g. improving food choices in canteens) alongside cognitive elements (e.g. education on nutrition) [42, 43]. In particular, when it comes to workplace nutrition and physical activity interventions, multilevel initiatives, combining environmental and cognitive elements, are shown to significantly impact factors such as absenteeism, work performance, workability, and productivity [42, 43].

Alongside these elements, young people also commonly viewed financial incentives, such as free/reduced gym memberships, as beneficial, especially when they could not afford them without having this as an employee benefit. There is some evidence corroborating the view that the use of financial incentives in the workplace leads to an increase in physical activity, but there are mixed results in relation to sustained outcomes, with some evidence showing increased activity in the long-term, whereas others showed a decrease [44].

### The role of relational factors

While the evidence for structural, environmental and cognitive interventions is robust, it is important to note that there is no one-size-fits-all solution. There is no single ‘perfect’ design, but rather a number of criteria that can improve the chances of intervention success, which often include tailoring the intervention to the workforce. This requires utilising the unique psychosocial assets of the workplace, particularly social support and organisational culture, ensuring management buy-in and employee involvement [43, 45]. These factors sit outside the interventions themselves but are essential to improve success and effectiveness, as participants in the research emphasised.

The findings highlight that where there were reports of some effective interventions to improve health behaviours (e.g. healthy food provision in offices), this had occurred because employer-employee communication allowed for a ‘safe space’ to voice opinions, and collaboration and co-creation of potential lifestyle interventions. Young people who reported more positive health behaviours also tended to be those who discussed having positive relationships with their managers and employers where they felt valued, supported, and like their voice and needs mattered. These considerations highlight the existence of key enablers of effective intervention implementation, relational factors. These are tied to the quality of relationships, communication, and employees’ sense of agency in the workplace [46, 47]. Previous evidence highlights that successful workplace health programmes are distinguished by strong support from senior leadership, a shared sense of responsibility among employees, voluntary, enjoyable participation, trust, and opportunities for co-creation of initiatives [45]. Conversely, a perceived divide between employees and management are reported as key obstacles to effective implementation of health interventions [45].

### Strengths and limitations of the research

Our research focused on a limited number of industries which were selected due to the high number of young adults they employ and distinct characteristics (early years education, social care, construction, food hospitality). However, these will not reflect working conditions of all young adults, and in particular we have not included industries with mainly desk-based roles who may have different challenges such as hybrid working. While we did not complete a formal inter-rater reliability assessment as part of the analysis process, regular discussions demonstrated a high degree of consensus among team members. The focus group format allowed fruitful and lively discussions with participants building on each other’s narratives. The discussion was aided by the use of Jamboard, an interactive whiteboard, where participants could add any further thoughts and insights around each research theme, in an anonymous way. We included a diverse mix of participants, ranging in age, ethnicity, gender, and location. However, given small numbers in each group, we were not able to make links between individual characteristics of participants and their ideas and experiences.

### Implications for future research and practice

Acknowledging that many of the recommendations for a healthy workplace are likely to increase costs for employers, at least in the short-term, challenges for the future are to determine which interventions are the most cost-effective, who should pay for these changes, and what aspects should be prioritised. Further research should explore employers’ decision-making around implementation of support for healthy behaviours, as well as the trade-offs they face between investing in health-improvement interventions and investing in other initiatives, including other employee benefits and overall renumeration. Understanding the extent of these trade-offs and how employers would respond to policy incentives designed to encourage further investment in employee health is critical. Employers are a key facilitator who can, with their policies and practices, help enable young adults to maintain healthy lifestyle.

## Conclusions

Our study demonstrates that young people and employers recognise the importance of work on health. Young people and employers share concerns about the negative impact of some aspects of work (e.g. long working hours or lack of facilities) but also the potential positive influence of work on young people’s health behaviours. While young people recognise the role of employers in supporting their health, they deem that individuals are ultimately responsible. Employers primarily emphasise their role in providing education and raising awareness about healthy lifestyles. Structural, environmental and relational factors are all important in ensuring that workplaces support young people’s health.

## Supplementary Information

Supplementary Material 1

## Data Availability

The datasets generated and/or analysed during the current study are not publicly available due to the sensitive nature of the research, but are available from the corresponding author on reasonable request.

## Footnotes

1 Young people who were in part-time further education or training, and spend the equivalent of full-time hours in the workplace were eligible.
