# Supplementary material for: From lunch breaks to late nights: a qualitative study of how workplaces can support healthy diet, physical activity and sleep behaviours in young adults
Source: BMC Public Health. 2025 Dec 17;25:4262. doi: 10.1186/s12889-025-25595-8 (PMC12709798; doi:10.1186/s12889-025-25595-8)
Supplement: Supplementary file 1 — Supplementary Material 1 [file 12889_2025_25595_MOESM1_ESM.docx]

Supplementary Material

**Supplementary Table 1**: Weighted demographic composition (%) of employees in the four selected UK Industries compared to the national employed population

|  |  | Early Years Education  (Sample n=115) | Social care  (n=680) | Construction  (n=900) | Accommodation & food services (n=811) | Total employed population (n=31,665) |
| --- | --- | --- | --- | --- | --- | --- |
| Age | 16-24 | 10.03 | 11.20 | 12.52 | 25.40 | 7.79 |
|  | 25+ | 89.97 | 88.80 | 87.48 | 74.60 | 92.21 |
| Sex | Male | 3.63 | 19.99 | 97.50 | 44.17 | 51.78 |
|  | Female | 96.37 | 80.01 | 2.50 | 55.83 | 48.22 |
| Highest Qualification | Degree or Equivalent | 30.66 | 24.81 | 8.95 | 19.65 | 39.99 |
|  | Other Higher Education | 8.02 | 9.97 | 3.95 | 5.26 | 7.13 |
|  | GCE A-Level or Equivalent | 39.89 | 25.68 | 39.88 | 26.61 | 20.17 |
|  | GCSE Grades A*-C or Equivalent | 12.73 | 22,62 | 23.75 | 20.42 | 15.99 |
|  | Other qualification | 1.38 | 8.45 | 9.40 | 15.11 | 6.11 |
|  | No qualifications | 0.88 | 4.46 | 11.06 | 8.63 | 4.57 |
|  | Missing | 6.43 | 4.01 | 3.01 | 4.31 | 6.04 |
| National Statistics Socio-economic classification | Higher managerial, administrative and professional occupations | 49.71 | 4.01 | 0.00 | 12.63 | 49.92 |
|  | Intermediate occupations | 49.39 | 0.00 | 0.00 | 0.00 | 13.14 |
|  | Small employers and own account workers | 0.91 | 3.68 | 54.60 | 8.78 | 9.50 |
|  | Lower supervisory and technical occupations | 0.00 | 9.84 | 14.24 | 23.80 | 5.75 |
|  | Semi-routine and routine occupations | 0.00 | 82.47 | 31.15 | 54.79 | 21.68 |

Notes: Data taken from the Labour Force Survey, April-June 2023. Full-time students and those not in employment are excluded. Data are weighted on the basis of sub-national population totals by age and sex to give estimates for the entire UK household population. Industry groups were based on the SOC2020 classifications. Early Years Education included SOC codes 1233, 2324, 6111. Social care work included 6135, 6136, 6137, 1232. Construction included codes 531, 532, 533, 815, 912. Accommodation & food services included 543, 9261,9263, 9264, 9265, 9266, 1221, and 1222.

**Discussion guide for focus groups with employers**

What does ‘healthy lifestyle’ mean to you? What are some of the key things that come to mind?

- *Is It physical, mental, or both? How do they interact?*

For this project, we’re honing in on nutrition, physical activity and sleep. In what way can work/employers in your sector play a role in supporting young employee’s health in these spheres?

- *Mental health support/ physical health support*
- *Are you currently experiencing any challenges in your workplaces?*

What are the particular challenges for maintaining a healthy lifestyle for younger employees working in your sectors and organisations? Particularly in relation to nutrition, physical activity and sleep

- *How do they manifest themselves?*
- *How do you collect data about it/monitor it?*
- *How different is this to people of other ages in your sector/organisation?*

What access to support or opportunities at work do young employees have to support a healthy lifestyle in your organisations?

- *Access to EAPs/OH/manager support/lifestyle apps/canteens*
- *Physical workplace, job design, nature of employment contract*
- *If not – why not?*
- *Are these different to what employees of other ages have access to?*
- *Where do they go to do support?*
- *Are any of these currently evaluated*

What additional types of support or resources would be most helpful in promoting healthy lifestyle behaviours for young employees in your workplace?

- *Are there specific areas (e.g., nutrition and physical activity) that you feel need attention?*
- *How would you prefer to receive support?*
- *How can we make sure any additional support or intervention is engaging and effective for younger workers?*
- *Consideration of health in recruitment and retention practices and, employee benefits offered.*

**Discussion guide for focus groups with young adults**

What does ‘healthy lifestyle’ mean to you? What are some of the key things that come to mind?

- *Is It physical, mental, or both? How do they interact?*

Do you feel like work affects your health?

- How does work fit within your life when it comes to health?
- Does it support it? Does it affect it negatively? Or is it a bit of both?
- Are there specific challenges you face in maintaining a healthy lifestyle while working?

For this project, we’re particularly interested in nutrition, physical activity and sleep. Do you think there are particular challenges for maintaining a healthy lifestyle for young people working in construction / early years / food / social care in relation to these three aspects?

- Are there things in your workplace environment that affect these aspects (e.g., kitchen facilities, time schedules, job design incl. the tasks you have to do at work, autonomy and agency over your work)?
- Do any specific aspects of work have an impact on health behaviours outside of work?

Do you think that work/employers have a role to play in employees’ health, again in relation to nutrition, physical activity and sleep?

- Why yes? Why not?

Do you think that young people have access to support and opportunities at work that support a healthy lifestyle?

- Yes, the things that support it are...
- No, the things that prevent it are...

Where do you go for support, guidance and advice around health at work? Is it helpful?

- What are the things that work well and that you’d want to see employers keep doing/do more of?

What (additional) types of support or changes would be most helpful in promoting healthy lifestyle behaviours in your workplace?

- If you were to design a workplace initiative to promote healthy lifestyle behaviours, what elements or features would it include?
- Are there specific areas (e.g., nutrition, physical activity, stress management) that you feel need attention?
- How would you prefer to receive support?
- How can we make sure this intervention is engaging and effective for young adults like yourselves?
